# Optimizing checkpoint inhibitors therapy for relapsed or progressive classic Hodgkin lymphoma by multiplex immunohistochemistry of the tumor microenvironment

**DOI:** 10.1002/cam4.2168

**Published:** 2019-05-08

**Authors:** Antonino Carbone, Annunziata Gloghini, Giancarlo Pruneri, Riccardo Dolcetti

**Affiliations:** ^1^ Centro di Riferimento Oncologico di Aviano (CRO) IRCCS Aviano Italy; ^2^ Department of Diagnostic Pathology and Laboratory Medicine Fondazione IRCCS, Istituto Nazionale dei Tumori di Milano Milan Italy; ^3^ University of Queensland Diamantina Institute, Translational Research Institute, University of Queensland Brisbane QLD Australia

**Keywords:** checkpoint blockade, classic Hodgkin lymphoma, immune escape, multiplex immunohistochemistry, resistance, tumor microenvironment

## Abstract

Immune checkpoint‐blocking antibodies have therapeutic activity against relapsed or progressive classic Hodgkin lymphoma (cHL), but Hodgkin Reed‐Sternberg cells can develop resistance to this therapy via multiple mechanisms. To improve the efficacy of immune checkpoint blockade, we need a more precise understanding of the immune escape mechanisms active in individual cHL patients, and this requires a detailed characterization of immune cell populations in the tumor microenvironment. These cell‐cell interactions can now be studied by multiplex immunohistochemistry coupled to digital image analysis. This method should allow the identification of actionable target molecules mediating resistance to immune checkpoint inhibitors in individual cHL patients, thereby favoring the implementation of personalized therapies.

A diagnosis of classic Hodgkin lymphoma (cHL) is based on the finding of neoplastic Hodgkin Reed‐Sternberg (HRS) cells within the heterogeneous cellular setting of a lymph node.^1^, ^2^ The tumor microenvironment (TME) includes reactive lymphocytes, eosinophils, granulocytes, histiocytes, macrophages, plasma cells, and mast cells.^2^ These cells express immunoregulatory molecules that serve fundamental roles in normal physiology, but are also involved in cancer cell growth, survival, and immune escape. This complex TME supports the survival of HRS cells through various cellular mechanisms, and HRS cells evade normal antitumoral immunity by expressing inhibitory ligands, resisting apoptosis, and inducing an immunosuppressive TME.^3^

Programmed cell death ligand 1 (PD‐L1) expression is invariably observed among at least a large fraction HRS cells in nearly all cases of cHL. PD‐L1 expression is driven by gains of chromosome 9p24.1, the locus that includes PD‐L1, PD‐L2, and JAK2. Gains at 9p24.1 directly increase the PD‐1L expression and JAK2 expression. Increased JAK2 may result in a heightened sensitivity of HRS cells to cytokine‐mediated JAK‐STAT signaling and thus even greater PD‐L1 expression due to cytokine‐mediated induction of the protein.^4^, ^5^ One mechanism of immune evasion involves signalling between PD‐L1, expressed by HRS cells, and its receptor programmed cell death 1 (PD‐1), expressed by immune cells. PD‐L1 in HRS cells binds PD‐1 on CD4 + T cells and CD8 + T cells, and suppresses T‐cell effector function. This so‐called PD‐1–PD‐L1 axis is a critical checkpoint that regulates the efficacy of T cell‐mediated immune responses, so blocking this pathway is the basis for cHL immunotherapy using checkpoint‐blocking antibodies (eg, nivolumab,^6^, ^7^ pembrolizumab^8^, ^9^). This strategy begins to be used when patients affected by cHL do not respond adequately to initial therapy (first‐line or second‐line treatments) or relapse.^10^ The therapeutic activity of nivolumab was recently shown in two clinical trials^6^, ^7^ of cHL patients who had failed to respond to autologous hematopoietic stem cell transplantation and brentuximab vedotin. On the basis of these trials, nivolumab was approved for relapsed or progressive cHL.^11^ The main mechanisms involved in cHL cell survival and immune escape are illustrated in Figure 1. First, HRS cells express high levels of PD‐L1, which binds its receptor PD‐1 on T cells and subsequently deactivates T‐cell antitumor function. Tumor cells also evade antitumor immune functions by encouraging the local infiltration of various immunosuppressive cells.^12^ For example, by secreting granulocyte‐macrophage colony‐stimulating factor, HRS cells stimulate the infiltration of myeloid‐derived suppressor cells (MDSCs).^13^ These cells suppress immune surveillance in cancer and inflammation.^14^ Immunosuppressive effects in the TME are also due to the accumulation of M2 macrophages, a subset of CD163 + macrophages that have anti‐inflammatory properties.^15^ A fraction of infiltrating CD4 + T cells are regulatory T (Treg) cells, which enhance immunosuppressive effects and whose presence is associated with inferior outcome.^16^ Furthermore, natural killer cells, whose function is to destroy diseased host cells such as HRS cells, have been reported to be defective in cHL patients.^17^ It is unknown what may be the contribution of these cells to the induction or the inhibition of clinical responses. Finally, another well‐characterized immune‐suppressive mechanism employed by HRS cells is the expression of the immune‐modulatory glycoprotein Galectin‐1. HRS cells invariably express Gal1 and Gal1 binding its ligands on T‐cells results in their apoptosis.^18^, ^19^

**Figure 1:**
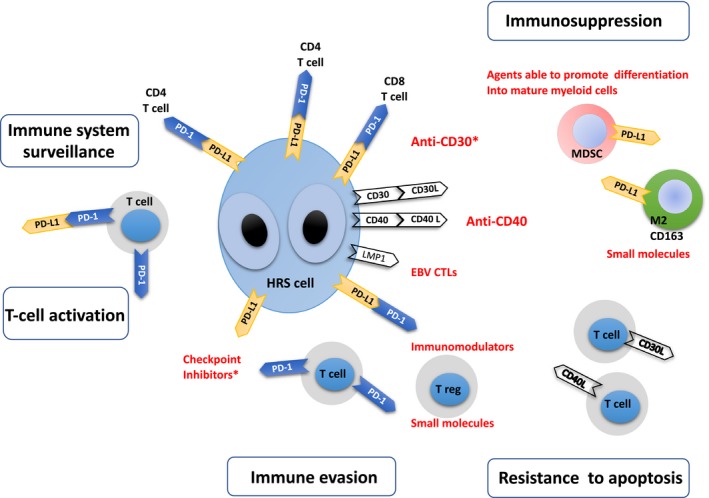
Mechanisms of tumor progression and tumor microenvironment‐mediated immune evasion in classic Hodgkin lymphoma (cHL). Left: Programmed cell death ligand 1 (PD‐L1) normally binds PD‐1 on T cells and regulates their activity. Centre: In cHL, PD‐L1 is also expressed by Hodgkin Reed‐Sternberg (HRS) cells. In these cells, PD‐L1 binds PD‐1 on CD4 + T cells and CD8 + T cells and suppresses T‐cell effector function. Regulatory T cells (Tregs) and the PD‐1: PD‐L1 pathway are both critical to terminating immune responses. Tregs lead to inhibition of the activity of conventional T cells. Right: Infiltration of the tumor microenvironment (TME) by myeloid‐derived suppressor cells (MDSC) and CD163 + M2 macrophages inhibit immune surveillance in cHL. Inflammatory and immune cells infiltrating the TME also express ligands (eg, CD30L and CD40L) that bind receptors on HRS cell membranes. In some cases, Epstein‐Barr virus infects the tumor clone, and the viral latent membrane protein 1 (LMP1) both augments HRS cell PD‐L1 expression and helps HRS cells resist apoptosis.^27^ In red, therapeutic agents targeting signals that allow HRS cells to evade immune surveillance and to resist apoptosis. Asterisks indicate U.S. Food and Drug Administration approved agents

Given the particularities of cHL, where a small number of tumor cells reside in close proximity to various types of immune cells, greater information on the spatial distribution of these cells is required. This need is even more important in the immunotherapy era to support therapeutic decision‐making. The choice of therapy for a particular patient should take into account not only the level of expression of the protein targeted by a therapy, but also the location and phenotype of immunosuppressive cells in the TME. In particular, it is important to know which secretory ligands and membrane‐bound molecules are being expressed by immune cells that are in proximity to HRS cells and that may be providing signals that allow HRS cells to resist apoptosis.

Hodgkin Reed‐Sternberg cells' ability to process and present antigens may also dictate their susceptibility to immunotherapy. In solid tumors, the response to immune checkpoint blockade requires tumor antigen presentation by HLA class I molecules on cancer cells to cytotoxic CD8 + T lymphocytes. In cHL, however, most tumor cells do not express HLA class I due to loss of beta2‐microglobulin.^20^ As revealed by the CheckMate 205 trial (ClinicalTrials.gov identifier: NCT02181738),^7^ HRS cell expression of β2‐microglobulin and HLA class I molecules was not predictive of the response to nivolumab.^21^ Intriguingly, HRS cell expression of HLA class II molecules was instead predictive of complete remission in the same study.^21^ This finding suggests that CD4 + T cells play a role in mediating the response to PD‐1 blockade via an alternative, HLA class II‐dependent mechanism. CD4 + T lymphocytes are a major component of the immune infiltrate of cHL; a detailed functional phenotyping and analysis of the spatial distribution of these cells may reveal the mechanisms of resistance to immune checkpoint blockade.

The identification of immune escape mechanisms should have therapeutic implications because the characterization of these mechanisms in individual cHL patients may guide the choice of immunotherapy or combination therapy. Achievement of this clinically relevant goal requires the detailed study of immunomodulatory proteins expressed by the different cell populations infiltrating the TME. This analysis is now possible using multiplex immunofluorescence or immunohistochemistry coupled to digital image analysis.^22^ This novel method uses three or more stains to detect multiple proteins simultaneously on the same tissue section (Figure 2 Panel A).^23^ The use of multiplexing for immune profiling is permitting the detection of multiple immunomodulatory molecules (eg, PD‐1 and PD‐L1) in single cells in histological specimens and the analysis of whether or not these molecules colocalize.^24^, ^25^ A multiplexing panel must be validated by standard immunohistochemistry for each of the selected antibodies (an example is shown in Figure 2 Panel B). Furthermore, to get insight into possible protein‐protein interactions, multiplex immunohistochemistry can be integrated with the in situ proximity ligation assay.^26^

**Figure 2:**
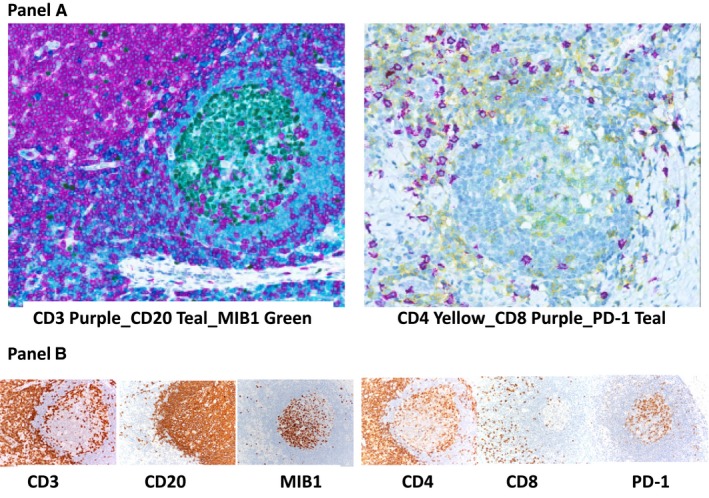
Panel A, Multiplex immunohistochemistry. Three stains can simultaneously detect different proteins in formalin‐fixed, paraffin‐embedded sections of a reactive lymph node. Left: Expression of CD3 (purple) in T cells, CD20 (teal) in B cells, and both CD20 and MIB1 (green) in a large fraction of germinal centre B cells, in different subcellular locations (CD20 in the membrane and MIB1 in the nucleus). Right: Expression of CD4 (yellow) in helper T cells, CD8 (purple) in cytotoxic T cells, and both CD4 (yellow) and programmed cell death 1 (PD‐1) (teal) in a large fraction of germinal centre T cells (merging into green). Panel B, Standard immunohistochemistry. Different tissue sections of a lymph node are stained with CD3, CD20, MIB1 and CD4, CD8, PD‐1. Left: Expression of CD3 (diffuse in the paracortical area and scattered in the germinal centre), CD20 (diffuse in the follicle mantle and scattered in the germinal centre), and MIB1 (restricted to germinal centre cells). Right: Expression of CD4 and CD8 (diffuse in the paracortical area). CD4‐positive cells are present in the germinal centre. A fraction of germinal centre cells also express PD1. Images were acquired with the Aperio ScanScope XT Virtual microscopy system and ImageScope Slide Viewing software (Leica Biosystems)

The fact that HRS cells use different mechanisms to escape antitumor immunity is currently limiting the efficacy of immune checkpoint blockade. The multiplex analysis of the TME could reveal which cells (eg, MDSCs, M2 macrophages, Tregs) and proteins (eg, PD‐L1, PD‐1) are limiting the efficacy of immunotherapy in individual cHL patients, and these results may guide the choice of a personalized treatment. The personalized combination of monoclonal antibodies, immunomodulators, and checkpoint inhibitors with mechanism‐based therapies may make HRS cells vulnerable to immune eradication. This approach has important basic scientific and translational implications, since it will enable future investigations into the mechanisms regulating immunity to cHL and will extend the perspective of optimizing immunotherapy for relapsed or progressive cHL and probably also for newly diagnosed disease.

## CONFLICT OF INTEREST

The authors declare no competing financial interests.
